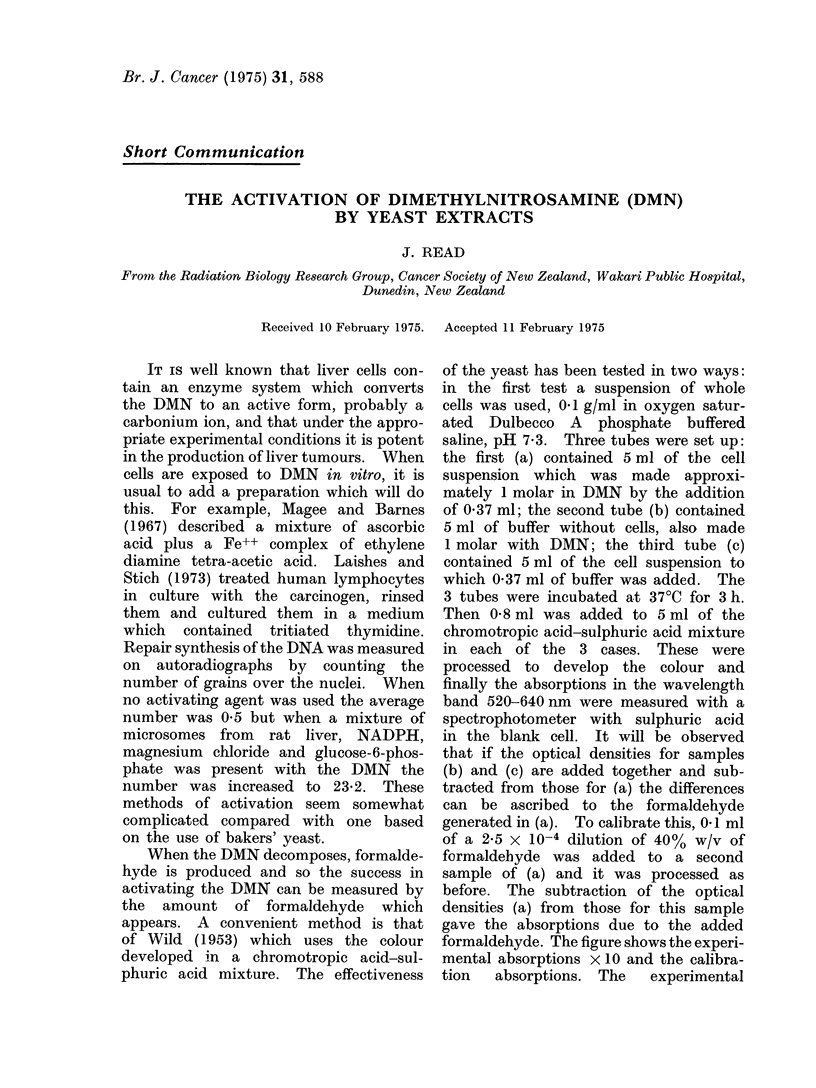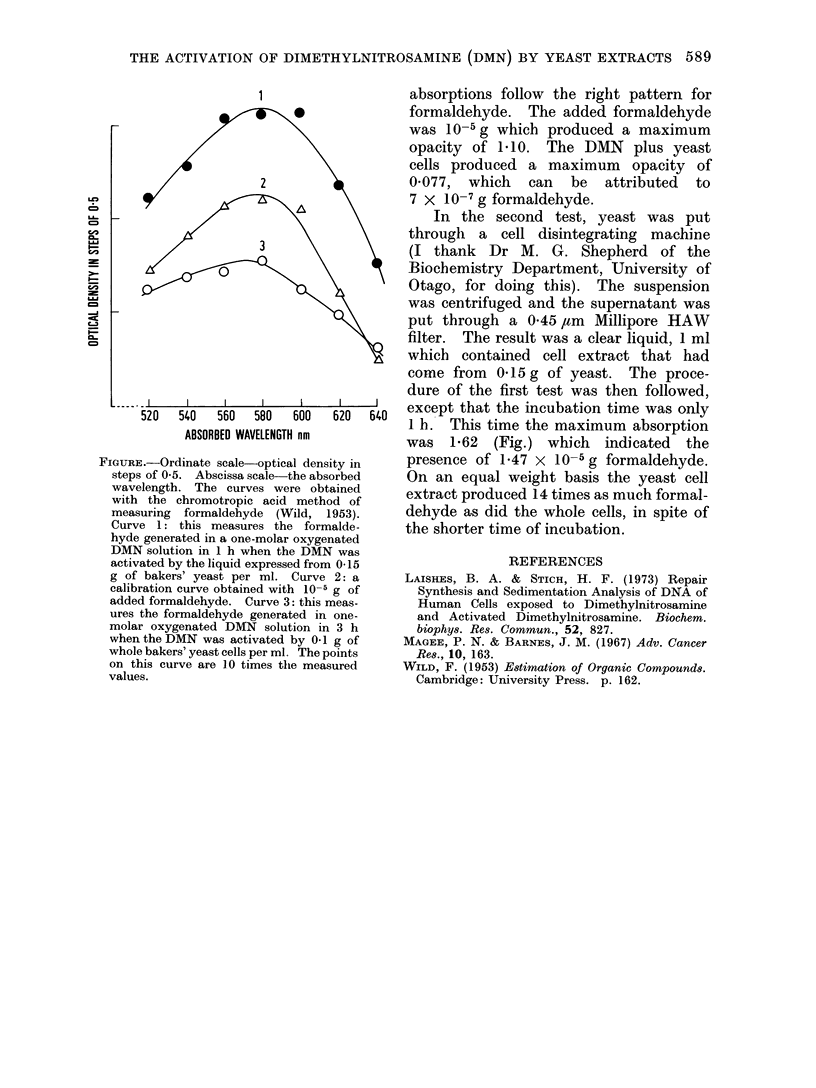# The activation of dimethylnitrosamine (DMN) by yeast extracts.

**DOI:** 10.1038/bjc.1975.101

**Published:** 1975-05

**Authors:** J. Read


*Br. J. Cancer* (1975) **31**, 588

### *Short Communication*

### THE ACTIVATION OF DIMETHYLNITROSAMINE (DMN) BY YEAST EXTRACTS

J. READ

*From the Radiation Biology Research Group, Cancer Society of New Zealand, Wakari Public Hospital, Dunedin, New Zealand*

Received 10 February 1975. Accepted 11 February 1975

IT IS well known that liver cells contain an enzyme system which converts the DMN to an active form, probably a carbonium ion, and that under the appropriate experimental conditions it is potent in the production of liver tumours. When cells are exposed to DMN *in vitro*, it is usual to add a preparation which will do this. For example, Magee and Barnes (1967) described a mixture of ascorbic acid plus a $Fe^{++}$ complex of ethylene diamine tetra-acetic acid. Laishes and Stich (1973) treated human lymphocytes in culture with the carcinogen, rinsed them and cultured them in a medium which contained tritiated thymidine. Repair synthesis of the DNA was measured on autoradiographs by counting the number of grains over the nuclei. When no activating agent was used the average number was 0·5 but when a mixture of microsomes from rat liver, NADPH, magnesium chloride and glucose-6-phosphate was present with the DMN the number was increased to 23·2. These methods of activation seem somewhat complicated compared with one based on the use of bakers' yeast.

When the DMN decomposes, formaldehyde is produced and so the success in activating the DMN can be measured by the amount of formaldehyde which appears. A convenient method is that of Wild (1953) which uses the colour developed in a chromotropic acid–sulphuric acid mixture. The effectiveness of the yeast has been tested in two ways: in the first test a suspension of whole cells was used, 0·1 g/ml in oxygen saturated Dulbecco A phosphate buffered saline, pH 7·3. Three tubes were set up: the first (a) contained 5 ml of the cell suspension which was made approximately 1 molar in DMN by the addition of 0·37 ml; the second tube (b) contained 5 ml of buffer without cells, also made 1 molar with DMN; the third tube (c) contained 5 ml of the cell suspension to which 0·37 ml of buffer was added. The 3 tubes were incubated at 37°C for 3 h. Then 0·8 ml was added to 5 ml of the chromotropic acid–sulphuric acid mixture in each of the 3 cases. These were processed to develop the colour and finally the absorptions in the wavelength band 520–640 nm were measured with a spectrophotometer with sulphuric acid in the blank cell. It will be observed that if the optical densities for samples (b) and (c) are added together and subtracted from those for (a) the differences can be ascribed to the formaldehyde generated in (a). To calibrate this, 0·1 ml of a $2 \cdot 5 \times 10^{-4}$ dilution of 40% w/v of formaldehyde was added to a second sample of (a) and it was processed as before. The subtraction of the optical densities (a) from those for this sample gave the absorptions due to the added formaldehyde. The figure shows the experimental absorptions $\times 10$ and the calibration absorptions. The experimental

FIGURE.—Ordinate scale—optical density in steps of 0·5. Abscissa scale—the absorbed wavelength. The curves were obtained with the chromotropic acid method of measuring formaldehyde (Wild, 1953). Curve 1: this measures the formaldehyde generated in a one-molar oxygenated DMN solution in 1 h when the DMN was activated by the liquid expressed from 0·15 g of bakers' yeast per ml. Curve 2: a calibration curve obtained with $10^{-5}$ g of added formaldehyde. Curve 3: this measures the formaldehyde generated in one-molar oxygenated DMN solution in 3 h when the DMN was activated by 0·1 g of whole bakers' yeast cells per ml. The points on this curve are 10 times the measured values.

absorptions follow the right pattern for formaldehyde. The added formaldehyde was $10^{-5}$ g which produced a maximum opacity of 1·10. The DMN plus yeast cells produced a maximum opacity of 0·077, which can be attributed to $7 \times 10^{-7}$ g formaldehyde.

In the second test, yeast was put through a cell disintegrating machine (I thank Dr M. G. Shepherd of the Biochemistry Department, University of Otago, for doing this). The suspension was centrifuged and the supernatant was put through a 0·45 $\mu$m Millipore HAW filter. The result was a clear liquid, 1 ml which contained cell extract that had come from 0·15 g of yeast. The procedure of the first test was then followed, except that the incubation time was only 1 h. This time the maximum absorption was 1·62 (Fig.) which indicated the presence of $1{\cdot}47 \times 10^{-5}$ g formaldehyde. On an equal weight basis the yeast cell extract produced 14 times as much formaldehyde as did the whole cells, in spite of the shorter time of incubation.

### REFERENCES

LAISHES, B. A. & STICH, H. F. (1973) Repair Synthesis and Sedimentation Analysis of DNA of Human Cells exposed to Dimethylnitrosamine and Activated Dimethylnitrosamine. *Biochem. biophys. Res. Commun.*, **52**, 827.

MAGEE, P. N. & BARNES, J. M. (1967) *Adv. Cancer Res.*, **10**, 163.

WILD, F. (1953) *Estimation of Organic Compounds.* Cambridge: University Press. p. 162.